# DiTeX: Disease-related topic extraction system through internet-based sources

**DOI:** 10.1371/journal.pone.0201933

**Published:** 2018-08-03

**Authors:** Jungwon Yoon, Jong Wook Kim, Beakcheol Jang

**Affiliations:** Department of Computer Science, Sangmyung University, Seoul, South Korea; Institute for Complex Systems, CNR, ITALY

## Abstract

This paper describes the web-based automated disease-related topic extraction system, called to DiTeX, which monitors important disease-related topics and provides associated information. National disease surveillance systems require a considerable amount of time to inform people of recent outbreaks of diseases. To solve this problem, many studies have used Internet-based sources such as news and Social Network Service (SNS). However, these sources contain many intentional elements that disturb extracting important topics. To address this challenge, we employ Natural Language Processing and an effective ranking algorithm, and develop DiTeX that provides important disease-related topics. This report describes the web front-end and back-end architecture, implementation, performance of the ranking algorithm, and captured topics of DiTeX. We describe processes for collecting Internet-based data and extracting disease-related topics based on search keywords. Our system then applies a ranking algorithm to evaluate the importance of disease-related topics extracted from these data. Finally, we conduct analysis based on real-world incidents to evaluate the performance and the effectiveness of DiTeX. To evaluate DiTeX, we analyze the ranking of well-known disease-related incidents for various ranking algorithms. The topic extraction rate of our ranking algorithm is superior to those of others. We demonstrate the validity of DiTeX by summarizing the disease-related topics of each day extracted by our system. To our knowledge, DiTeX is the world’s first automated web-based real-time service system that extracts and presents disease-related topics, trends and related data through web-based sources. DiTeX is now available on the web through http://epidemic.co.kr/media/topics.

## Introduction

Concerns about disease-related issues have increased due to the year-after-year appearance of diseases such as MERS, Zika virus, Avian Influenza, and Ebola virus. Developing a vaccine to prevent these diseases consumes considerable amount of time and very high sums of money. In addition, Centers for Disease Control and Prevention (CDCs) have existed in Europe and USA since the end of World War II and many other countries have established their own CDCs respectively, such as Korea Centers for Disease Control and Prevention (KCDC) and Chinese Center for Disease Control and Prevention (CCDC), where people can view disease-related information online. These CDCs surveil infectious diseases according to levels of priority, and the most dangerous diseases such as Avian Influenza, Ebola virus, and Malaria take a short lead time to transmit information to people than the less dangerous diseases. However, the CDCs rely on the centralized management system, which means a lead time is required to collect and produce disease outbreak statistics [1,2], which makes it difficult to respond instantly to new disease outbreaks [3–5].

People use various information channels to search for disease-related topics. Large portal sites such as Google, Yahoo, and Baidu provide users with real-time information and query ranking through Internet-based news stories. Real-time query ranking is the method by which the user search of the most frequently searched words go up to the top rank. However, this function cannot expose any disease-related topics if malicious users intentionally search a particular word multiple times to raise its ranking [6,7]. Moreover, Internet news articles offer information on a variety of topics such as politics, entertainment and fashion, and they often miss or are rarely interested in disease-related topics.

In addition to large portal sites, Social Network Services (SNSs) are also playing a great role as information channels. In particular, Twitter is one of the largest SNSs, on which many people communicate each other. Because of its importance, many researchers are conducting studies to analyze trends that are the most discussed, to monitor earthquakes [8,9] or to collect political opinions [10–12]. They are also working on collecting information about people's health by analyzing tweets (a tweet is a sentence on Twitter) [13–16]. Although these tweets are limited to 280 characters in USA and 140 characters in Korea, Japan, and China since 2017, it is difficult to extract information related to disease [17–22] because they have various attributes such as irregular grammar, repetition of meaningless text, and lots of advertising spam [3].

Many researchers have developed and run disease surveillance systems to solve these problems. For example, HealthMap [23] provides users with valuable disease-related information using a visualization service comprising news reports and user input and displays disease risk levels on a map. BioCaster [24] has collected various Internet-based data and linked user data with geodata and disease data through user participation filtering and BioCaster ontology. EpiSPIDER [25] also collects Internet-based data and provides users with disease-related information. These services are collecting various Internet-based data in multiple languages; however, they do not provide information on disease-related topics and disease-related trends. Thus, analyzing and detecting new disease outbreaks using these platforms is difficult.

Thus, we have developed the world's first automated web-based real-time disease-related topic extraction system, called to DiTeX, to solve these problems. DiTeX extracts disease-related topics from news and SNS in the Web environment. DiTeX is a service that has been in operation since August 14, 2017, free for all users, and view disease-related topics at any time. DiTeX extracts disease-related topics through fully automated processes, provides relevant information like news articles and tweets, and stores collected topics. Our system evaluates the importance of disease-related topics through an effective ranking algorithm after collecting disease-related information from news and SNSs. These information are automatically collected using disease-related search keywords. Online news sources interrupt the extraction of disease-related data by repeatedly using major keywords. SNS data is generated by many unspecified users, making it very difficult to extract accurate information owing to noise such as typos, repetition of meaningless words, and spam [3]. We successfully extracted data with minimal duplication using string similarity checks and converted incomplete sentences into complete sentences through open-source Natural Language Processing (NLP) to facilitate disease-related topic extraction. Through DiTeX, people can search for and retrieve disease-related topics according to a user’s selected date, and receive additional information on disease-related topics. In addition, DiTeX provides user-friendly interface exploiting effective visualization techniques such as disease-related trend graphs and a word cloud.

## Materials and methods

### Related works

There have been many studies and services that automatically collect unstructured and irregular data and extract disease outbreak information for users. Disease surveillance systems collect data from various web-based resources and extract only highly-relevant data through text algorithms, and then uses this data to display information such as disease routes, occurrence status, and risk. BioCaster [24], developed by Collier, is a project that ran from 2006 to 2012. BioCaster collects data from Internet news articles, public health workers, and users, and applied various filtering methods along with BioCaster ontology. In addition, BioCaster uses text mining technology to perform real-time tracking what is occurring and what is and likely to occur. EpiSPIDER [25] collects news, Twitter, and WHO articles and combines these with geographic data to provide users with information about diseases. EpiSPIDER also provides users with a map interface and a word cloud to show users what topics are most active, and various filtering functions that allow users can find the information they want. Finally, HealthMap collects and visualizes disease-related data from the World Health Organization (WHO), Google News, and validated official alerts. HealthMap [23] extracts only disease-related data, links between diseases and regions, measures the risk of disease, and visualizes those risks of diseases through color coding. HealthMap collects the data of 87 disease categories and 89 countries; the accuracy of the HealthMap disease classifier is 84%.

Many researchers have researched to extract health-related information through web-based data [26–28]. Yingjie Lu et al. extract and categorize the most active topics related to health in online communities [26]. They collect data on health-related social media services and categorize them into five clusters. As a result, their results show an average accuracy of 83.5%. Kyle W. Prier et al. define health topics through Twitter [27]. They categorize topics that are relevant to tobacco and define words that are relevant to each topic. They analyze the association between topics and words, and find the most relevant word set. Jiang Bian et al. analyze Twitter using NLP and Machine Learning [28]. They argue that they could extract health-related topics from Twitter although Twitter is difficult to analyze because of the huge-level of noises.

Some papers [23–25] present service systems that track diseases when disease-related events occur, but users cannot find comprehensive information on disease-related topics unfortunately, while DiTeX focuses on the extraction and production of disease-related topics. Other papers [26–28] study methodologies that extract health-related information through web-based data, while DiTeX focuses on both the service system and the methodology. It also focuses on disease rather than health. We believe that DiTeX may help people to capture newly emerging diseases and a variety of disease-related information. DiTeX can analyze disease-related trends over time and extract real-time information on disease-related topics. To our knowledge, DiTeX is the world’s first automated web-based real-time service system that extracts and presents disease-related topics, trends and related data through web-based sources. DiTeX collects information from the news and Twitter which are public data. DiTeX complied with the terms of service for NAVER News API (https://developers.naver.com/products/terms/) and Twitter search API (https://twitter.com/ko/tos).

### Ranking algorithm

While many researchers have been working hard to extract topics that people are interested in, many unnecessary attributes of Internet-based data such as repetition of the same meaningless content and writing for marketing purposes are hampering the topic extractions. To solve this problem, many researchers have proposed various ranking algorithms. Term Frequency (TF) [29], one of the most widely used techniques in Information Retrieval (IR) and text mining, is a technique for extracting the most frequently mentioned words in a document. However, since TF assigns equal value to all words, it is not an ideal method as it interprets repeated meaningless words as important. Researchers have proposed various ranking algorithms such as Term Frequency-Inverse Document Frequency (TF-IDF) [36], SMART weighting scheme [32], BM25 [33], Robertson-Sparck-Jones weighting [34], INQUERY [35], and Combined Component Approach (CCA) [37].

The ranking algorithm applied to our proposed system is the CCA algorithm. CCA was developed by Humberto and Marcos and is based on Genetic Programming (GP) [38]. GP is a technology that was proposed by Koza to improve the Genetic Algorithm (GA), a technology based on natural selection. Their research shows that average accuracy of the CCA algorithm improved by 32.86% than other ranking algorithms such as TF, TF-IDF, SMART, BM25, and INQUERY. The components required for their algorithms are shown in Table 1. The ranking algorithm of CCA is defined as:

$$CCA=\left((99.09+t_{09})+(\left(\left(t_{06}\times t_{08})\times (t_{05}\times ((\left(\left(t_{06}\times t_{08})+(t_{07}+t_{08}\right))\times (t_{10}\times t_{01}\right))\right))+(\left(t_{06}\times t_{08})\times (t_{05}\times (\left(\left(t_{02}\times t_{04})+(t_{07}+t_{08}\right))\times (t_{10}\times t_{01}\right))\right)\right)\right))+(\left(t_{10}\times t_{01})+(\left(t_{06}\times t_{08})\times (t_{05}\times (\left(\left(t_{07}÷t_{03})+(t_{07}+t_{08}\right))\times (t_{10}+t_{01}\right))\right))\right)$$ (1)

**Table 1:** Units for Combined Component Approach (CCA).

Id	Formula	Description
*t*_01_	*tf*	Number of times a term occurs in a document [29]
*t*_02_	1 + log(*tf*)	Natural logarithm of *tf* [30]
*t*_03_	$0.5+\frac{0.5+tf}{\max\;tf}$	*tf* factor normalized by maximum *tf* [29,31]
*t*_04_	$\frac{1+log(tf)}{1+\log(\mathrm{avg}\;tf)}$	Part of SMART weighting scheme formula [32]
*t*_05_	$\frac{{(k}_{1}+1)\times tf}{{(k}_{1}((1-b)+b\times dl/avg\;dl)+dl)+tf}$	Part of Okapi BM25 ranking formula [33] (*k*_1_ = 1000)
*t*_06_	$\log\left(\frac{N}{df}+1\right)$	An alternative to Inverse document frequency (*idf*) [30]
*t*_07_	$\log\left(\frac{N-df+0.5}{0.5}\right)$	A variation of the Robertson Sparck Jones weight [34]
*t*_08_	$\log\left(\frac{N-df}{df}\right)$	A probabilistic inverse collection frequency [29]
*t*_09_	$\frac{log\frac{N+0.5}{df}}{\log\;N+1}$	Part of INQUERY formula [35]
*t*_10_	$\frac{1}{\sqrt{\sum_{i=0}^{2}w_{i,j}^{2}}}$	Cosine normalization where $w_{i,j}^{2}$ is *t*_01_ × *t*_06_ [29]
*t*_11_	*dl*	Document length (in bytes) normalization [31]

We examine how accurate CCA is to extract disease-related topics from news and SNS compared to other algorithms by captured disease-related incidents in Section 5.

### System architecture

**Fig 1:**
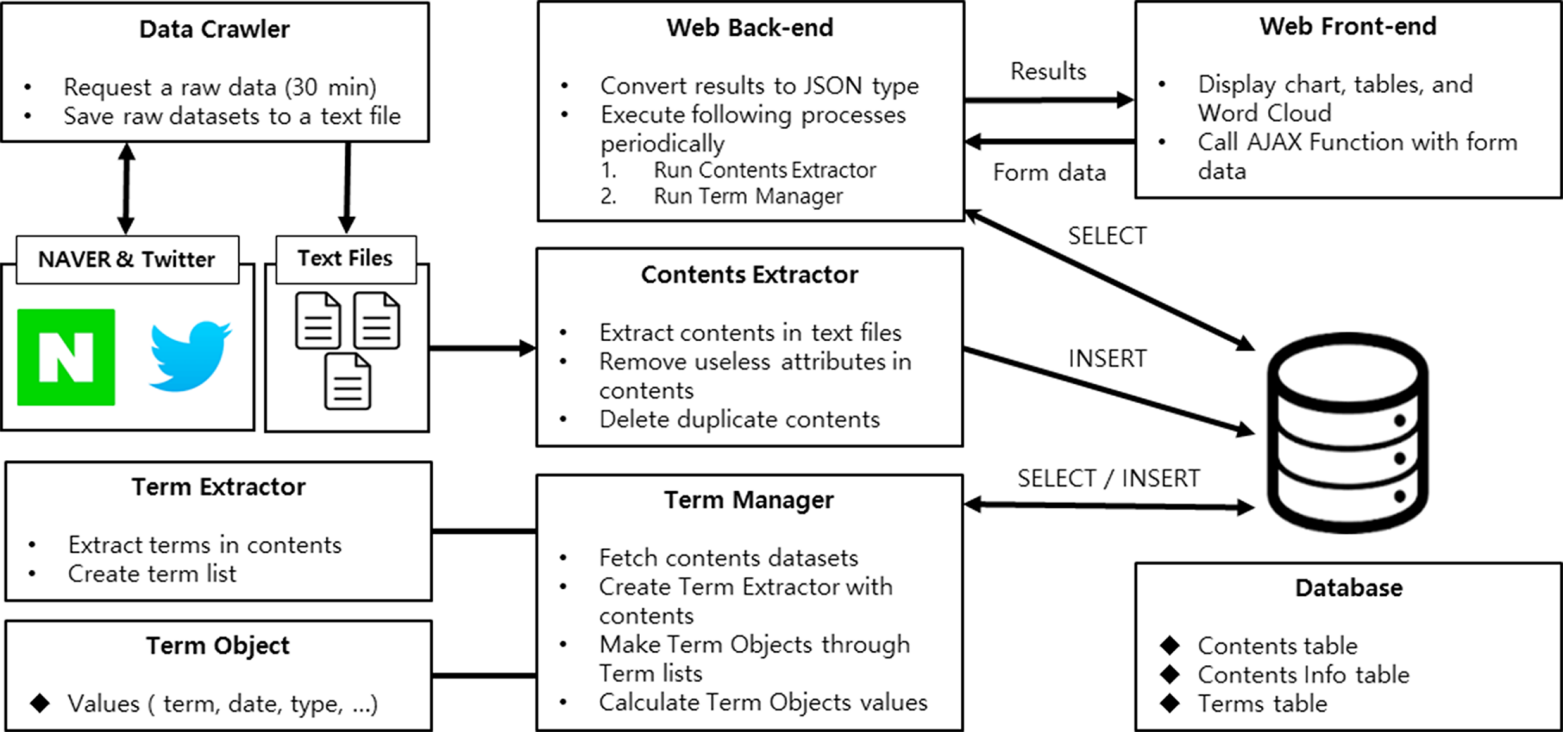
System architecture.

The specifications of DiTeX are as follows: the server was Windows Server 2016 Essentials x64, and the CPU is an Intel® Core ™ I7-4790 with 8 GB of RAM. DiTeX consists of a total of eight modules (see Fig 1). The data crawler collects news and SNS data. The back-end web changes the shape of the data according to the front-end web and performs processing to insert the collected data into the database. The content extractor removes the useless attributes and deletes duplicate data before storing final data in the database. The term manager retrieves the data from the database and creates the term extractor and term objects. Front-end web performs the role of managing view pages and visualizing disease-related data. Finally, the database was PostgreSQL 9.6, a relational database management system (RDBMS) [39]. The database consisted of the contents table, contents info table, and terms table. The contents table is a repository for storing data from the content extractor, and the contents info table stores data from the term manager. The terms table manages the data in term manager.

### Data crawler

Data Crawler collects 100 articles and tweets per hour based on a search Application Programming Interface (API) [40] provided by NAVER [41] and Twitter. The Search API is a REpresentational State Transfer (REST) API [42] structure that sends a specific Uniform Resource Locator (URL) to JavaScript Object Notation (JSON) [43] in the GET response of the Hypertext Transfer Protocol (HTTP) [44]. JSON is a lightweight text data type that consists of a single pair of name and value, used in various computer language environments. The Data Crawler stores a text file with a name that includes the date of the data collected from this technique and the search keyword.

Table 2 shows the search keywords collected by the data crawler. These are the most commonly used words on the detail page that describe the statutory infectious diseases and infectious diseases designated by the KCDC.

**Table 2:** List for disease related search keywords.

ENGLISH	KOREAN	ENGLISH	KOREAN
Chicken pox	수두	Fever	발열
Mumps	유행성이하선염	Cough	기침
Thrombocytopenia syndrome	중증열성혈소판감소증후군	Headache	두통
Japanese encephalitis	일본뇌염	Chills	오한
Vibrio vulnificus sepsis	비브리오패혈증	Myalgia	근육통
Legionella’s	레지오넬라증	Abdominal pain	복통
Scrub typhus	쯔쯔가무시증	Diarrhea	설사
Nephrotic syndrome	신증후군출혈열	High fever	고열
Leptospirosis	렙토스피라증	Hemorrhage	출혈
Influenza	인플루엔자	Infection	감염
Scarlet fever	성홍열	Arthralgia	관절통
Hepatitis C	C형간염	Inflammation	염증
CRE	카바페넴내성 장내세균속균종 감염증	Vomiting	구토
Hepatitis A	A형간염	Disease	질병
Syphilis	매독	Illness	질환
Streptococcus pneumoniae	폐렴구균	Syndrome	증후군
Malaria	말라리아	Communicability	전염
MERS	중동 호흡기 증후군	Epidemicity	유행성
Zika virus	지카 바이러스	Symptom	증상
Avian influenza	조류 인플루엔자	Vaccine	백신
Ebola virus	에볼라 바이러스	Incubation period	잠복기
Virus	바이러스	Cold	감기
Detection	검출	Influenza	독감
Prevention	예방	Influenza	인플루엔자
Disinfection	방역	Germ	세균
Definite diagnosis	확진	Bacteria	박테리아
		Occur	발병

### Back-end web

The back-end web is based on Spring 4 [45] and Jetty [46]. In addition, Model View Controller (MVC) [47], one of the computational design patterns, was applied. The main task was to convert the data requested by the front-end web into JSON data. The back-end web executed the modules (contents extractor, term manager, term extractor, and term object) that we set up at a specific time in the job scheduler, which operated periodically. The processes performed by the modules were as follows:

First, the content extractor retrieved the data collected by the data crawler and extracted only sentences from the data. The content extractor removed any unnecessary elements from the extracted sentences (URL, HTML tags, retweets, etc.). We used regular expression to remove unnecessary elements and change them to blank. Since these sentences were redundant and likely to be similar, we checked the similarity between sentences using Sift4 [48]. Sift4 is a string distance algorithm inspired by Jaro-Winkler [49] and the longest common subsequence principle [50]. We used Sift4 to extract the unique sentences that allow for the removal of duplicate and similar sentences. The content extractor stores these complete sentences in content table of the database. The content table consists of date collected, data type (News, SNS), and sentences.

Second, the term manager retrieved the sentences stored in the database by the content extractor. The term manager creates the term extractor and passes one statement to it. Following this, the term extractor extracts words from a sentence. The technique for extracting words was OpenKoreanTextProcessorJava [51]. OpenKoreanTextProcessorJava was developed by mixing JAVA [52] and Scala [53] and is the most widely used Natural Language Processing (NLP) technique in Korea. It has the largest Korean corpus and is constantly adding new words. The term extractor performed the generalization and tokenization of sentences. Generalization correctly changes misspelled words in a sentence. Through this process, we could obtain sentences composed of correct words. When this modified sentence performed a tokenization function, it delivered a list of tokens consisting of words/tags. We only collected from this list if the length of the word was greater than 1 and the word was a noun.

Third, term object refers to the object of extracted words. The term manager has term object list and creates new term object when a new word is found. After collecting the words of all sentences, the term manager applied the ranking algorithm to the term object list, and the value calculated from the CCA.

Finally, after calculation of the entire term object list was completed, the term manager and term object data were stored in database.

### Front-end web

The front-end web is the part of our system that visualizes disease-related data for users. Here, we used JQuery to request the information the users wanted from our system. JQuery [54] uses Asynchronous JavaScript and XML (AJAX) [55] to send data to and from the server to provide information for the dates requested by the user. The data received from the server is in JSON format, and the front-end web uses this data to create charts, tables, and word clouds. We used dataTables.js [56], D3.js [57], and chart.js [58] to provide visualization to users. DataTables.js is a library that creates a ranking table of disease-related topics and D3.js is a library that creates word clouds through which users can view multiple words. Chart.js provides the ability to create various charts, and we provided a trend of disease-related topics through the line chart.

## Result

**Fig 2:**
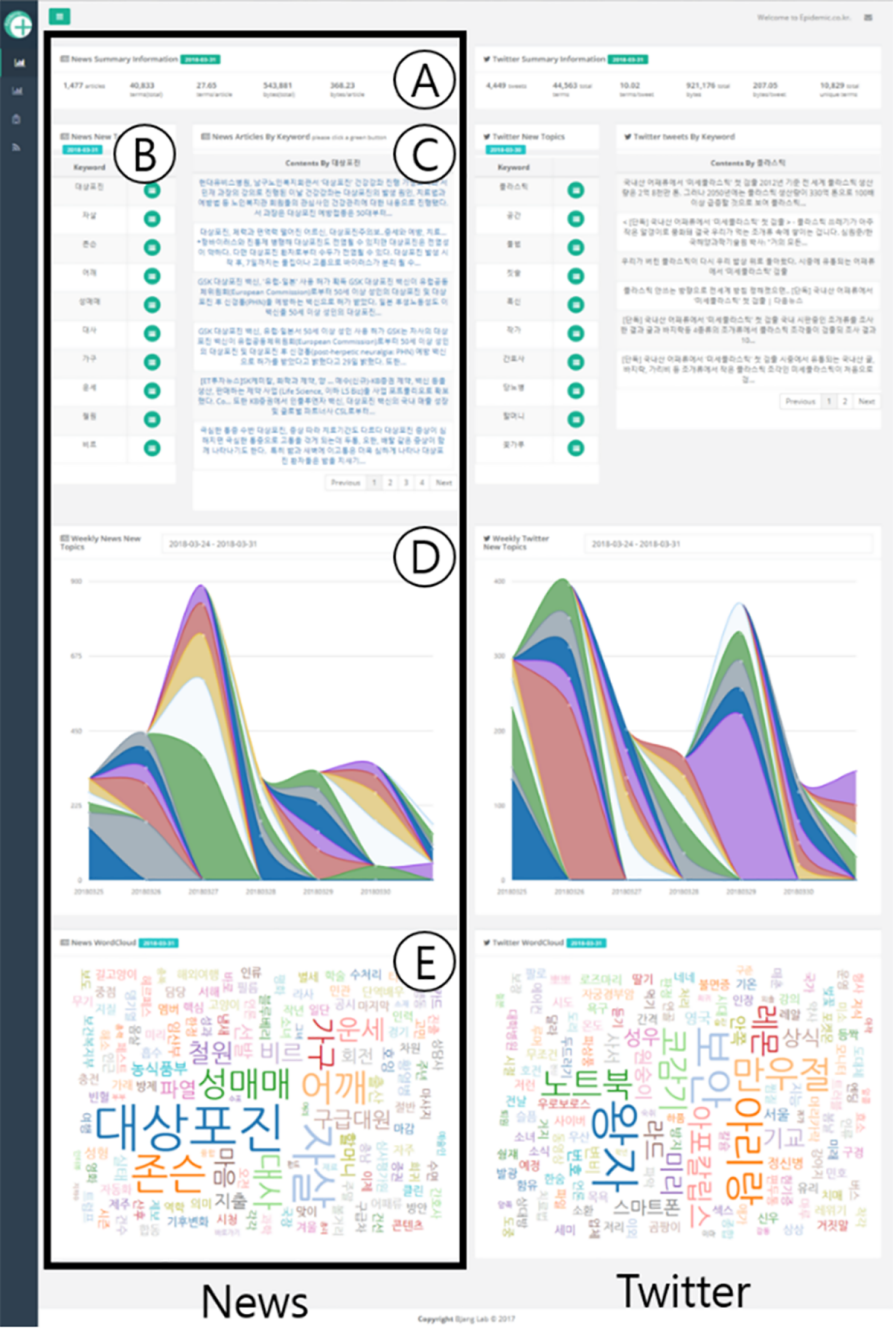
“Topics” page description.

Fig 2 is a full-screen view of the "Topics" page (http://epidemic.co.kr/media/topics), where users can always find disease-related topics for a desired date. "Topics" shows News and Twitter's disease-related topics and consists of four parts. Fig 2A outlines the data we collect, and Fig 2B is disease-related topics extracted from News or Twitter. Click on the topics in Fig 2B to see the articles or tweets associated with this topic in Fig 2C. Fig 2D is the trend graph of disease-related topics that depicts disease-related topics over a period of time. Finally, Fig 2E is the word cloud that helps users to grasp disease-related topics at a glance.

Fig 2A provides the comprehensive view of the data collected (from the left: the total number of collected data, the total number of extracted words, the average of the extracted words, the total bytes, the average bytes, and the number of unique words stored in the database). We can see through this part that news is more redundant than SNS information. The number of data collected for news and SNS was the same; however, the number of news articles after back-end web processing was greatly reduced. Although there was no difference in the number of words registered in the database (despite the difference in the total number of data and the number of extracted words on average), the news repeatedly uses the same words and SNSs use a variety of words. Fig 2B shows the top 10 disease-related topics with the highest CCA. With this function, we confirmed that the sources of SNSs information were often unclear, whereas the sources of news information were clear. This is because the indirect topics on SNSs consistently ranked highly. For example, on February 7, 2018, news returned data in the Top 10 for "Noro virus.” However, SNSs showed the same return for "virus," owing to SNS users mentioning "virus" more often than "Noro virus". Fig 2E is a word cloud created using the top 200 disease-related topics. The higher the CCA, the larger the topic size; thus, users can see what the most important information. News sources have more words for disease names, whereas SNSs have many words to describe symptoms. This occurs because SNS users generate data based on their personal experiences.

To extract newly emerging disease-related topics quickly, we prepare another page called to “New Topics”, which extracts newly appearing disease-related topics compared to those of the previous day. Its interface is similar to "Topics" page’s, but the details of Fig 2B, Fig 2D and Fig 2E are different. Fig 2B lists the topics that present new disease-related topics or a sharp rise in the CCA compared with previous data. Fig 2D is a trend graph of new disease-related topics, which confirms when that topic became active. Finally, Fig 2E is a word cloud created using these new topics. Users can check newly emerging disease-related topics through our "New topics" page quickly.

## Discussion

We compared the rankings of disease-related topics extracted through the CCA with those of other ranking algorithms. Finally, we show disease-related information on the "Topics" and "New topics" pages by date, thus proving the efficacy of our system.

Table 3 lists the disease-related topics for the TF, TF-IDF, TF-IDF (log), SMART, INQUERY, and CCA algorithms. Egg and pesticide contamination denotes the accident in which various pesticides in eggs have exceeded the regulation standards in Europe and Korea. “Ham Sausage” denotes the incident involving ham and sausage made of German and Dutch pork being the main cause of hepatitis. Finally, “Noro virus” denotes the case that occurred at the Pyeongchang Olympics in Korea. All cases are good examples by which to judge the performance of the topic extraction of CCA for incidents that have been reported globally. Therefore, we compared the topic extraction rates of five ranking algorithms for both news and SNS sources.

**Table 3:** Topics ranking about events.

	Aug 15, 2017 Egg Pesticide		Aug 24, 2017 Ham Sausage		Feb 8, 2018 Noro virus	
	News	SNS	News	SNS	News	SNS
TF [26]	3	282	15	87	22	134
TF-IDF [27]	1	103	3	46	6	55
TF-IDF (log) [27]	1	172	12	68	13	72
SMART [28]	1	111	12	57	12	59
INQUERY [31]	9	513	51	133	29	122
CCA [32]	3	53	1	32	3	45

All algorithms except INQUERY are excellent for extracting disease-related information from news sources. However, the difference in performance of ranking algorithms for SNSs was significant. CCA showed all events in 50th, but other algorithms were often larger than that. Among the ranking algorithms apart from CCA, the algorithm with the best performance was TF-IDF. TF-IDF can capture disease-related topics from SNS at a higher rank. TF-IDF is an older algorithm; however, it still performs well when extracting disease-related information from SNSs. On the other hand, CCA shows even better performance than TF-IDF. For “Egg Pesticide” contamination, TF-IDF ranked 103^rd^ on SNS, whereas CCA ranked 53^rd^—an increase rate of approximately 50%. Thus, CCA shows excellent extraction rates for disease-related topics from both news and SNS sources.

We use four evaluation metrics to evaluate the performance of DiTeX: Rand statistic, Jaccard coefficient, Fowlkes and Mallows (FM) index [59], and Odds Ratio [60], which are measurement techniques that can show the accuracy and possibility of capturing the events. The four metrics are defined as follows:

$$Rand\;statistic:\;R=\frac{TP+TN}{TP+TN+FP+FN}$$ (2)

$$Jaccard\;coefficient:\;J=\frac{TP}{TP+FP+FN}$$ (3)

$$FM\;index:\;FM=\frac{TP}{\sqrt{TP+FP}+\sqrt{TP+FN}}$$ (4)

$$Odds\;Ratio:\;OR=\frac{TP*TN}{FP*FN}$$ (5)

where TP is the number of news and SNS data related to the event that occurred, TN is the number of news and SNS data that are not related to events that have not occurred, FP is the number of news and SNS data related to events that have not occurred, and FN is the number of news and SNS data that is not related to the event that occurred. TP and TN are “good topic”, and FP and FN are “bad topics”. The Rand statistic in statistics is used to measure the similarity of two datasets such as “good topic” and “bad topic”. This means that the Rand statistic can determine the accuracy of capturing the topics in our DiTeX. The Jaccard coefficient in statistics is a measurement method of comparing the similarity and diversity of datasets. In other words, we can use the Jaccard coefficient when we analyze the accuracy that DiTeX captures “good topic” and “bad topic”. The FM index is also a measurement technique that determine the similarity between two datasets. Finally, the Odds Ratio is a measure of association between “good topic” and “bad topic”. That is, the Odds Ratio indicates the possibility of DiTeX to capture “good topic” versus “bad topic”.

**Fig 3:**
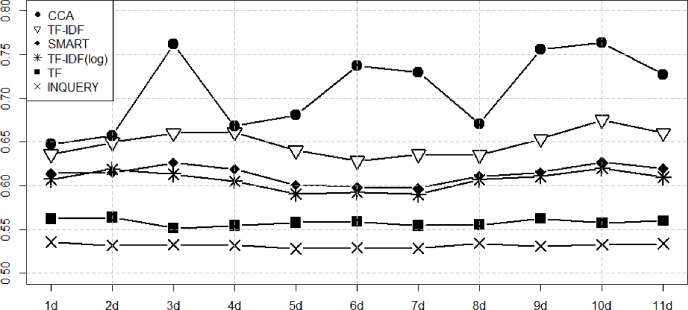
Performance measures of ranking algorithms using the Rand statistic from March 15 to March 25, 2018.

**Fig 4:**
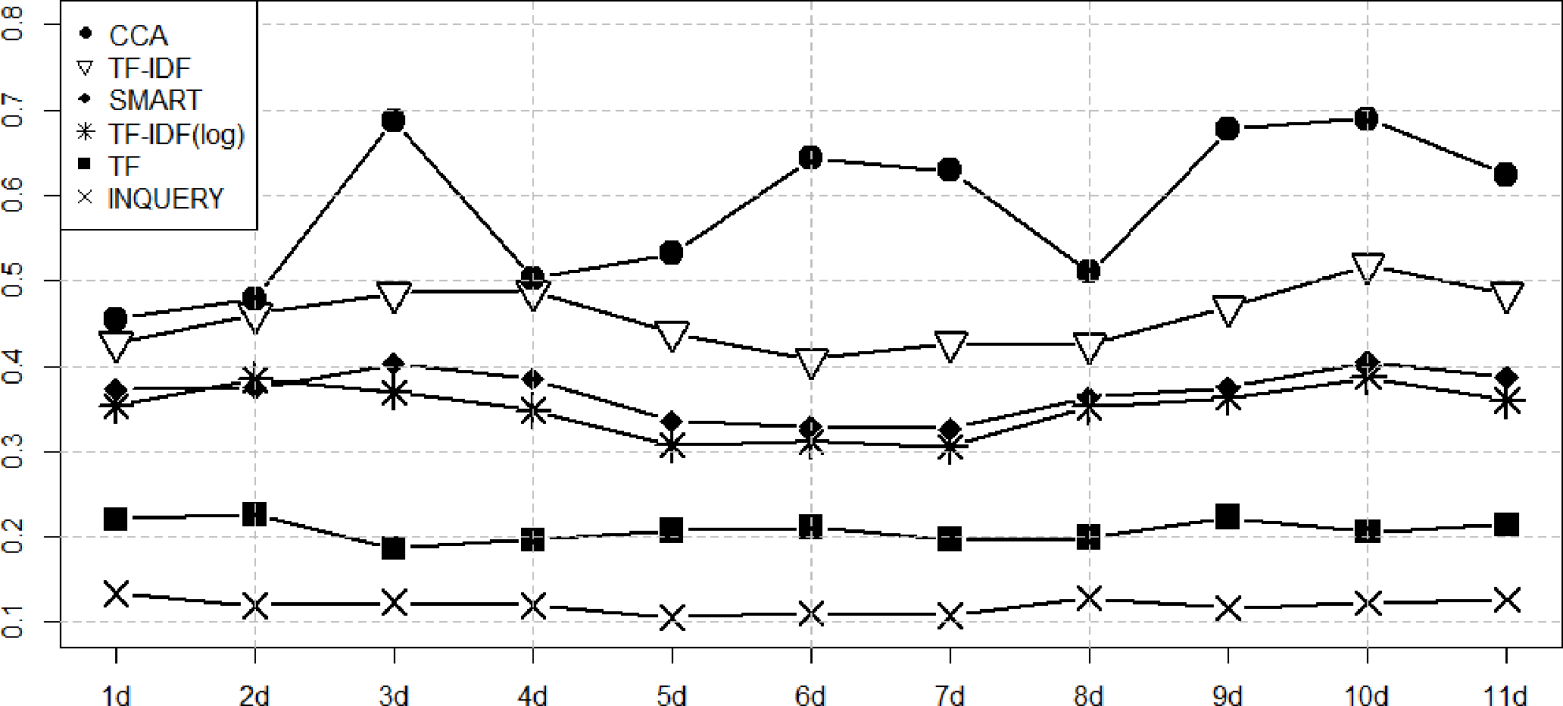
Performance measures of ranking algorithms using the Jaccard coefficient from March 15 to March 25, 2018.

**Fig 5:**
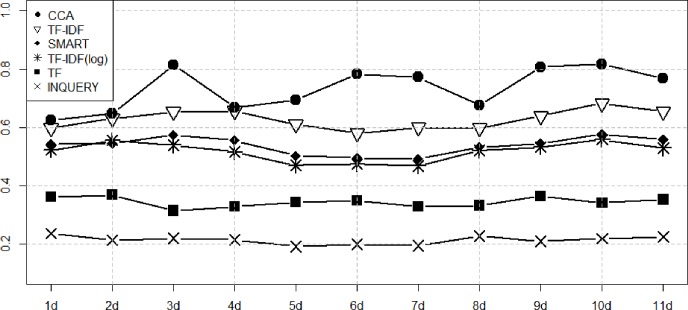
Performance measures of ranking algorithms using the FM index from March 15 to March 25, 2018.

**Fig 6:**
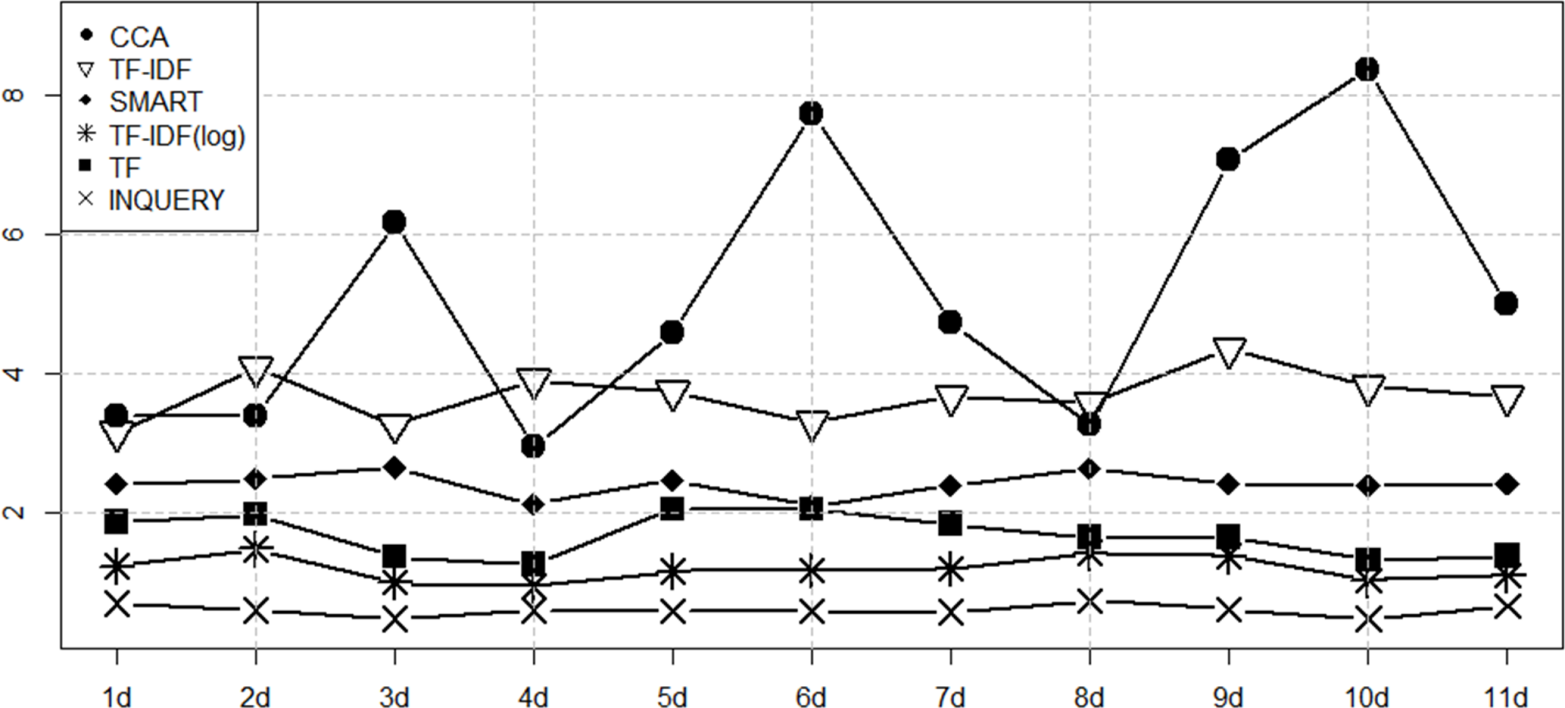
Performance measures of ranking algorithms using the Odds Ratio from March 15 to March 25, 2018.

Figs 3–6 show the performance of the ranking algorithms from March 15 to March 25, 2018 for the Rand statistic, Jaccard coefficient, FM index, Odds ratio. CCA shows significantly better performance than other ranking algorithms in all performance analyzes. In Fig 3, TF-IDF shows R = 0.649 on average, CCA is R = 0.709. In Fig 4, TF-IDF is on average J = 0.458, CCA shows J = 0.585. In Fig 5, TF-IDF shows FM = 0.627 on average, CCA is FM = 0.734. In Fig 6, TF-IDF shows OR = 3.683 on average, CCA shows OR = 5.153. CCA shows better performance than TF-IDF, which was the second best performance in Table 3, in all analyzes.

Table 4 lists disease-related topics captured by our system; specifically, our system can extract at least two disease-related topics on a monthly basis, capturing both domestic and international disease-related events. The increase in disease-related topics since March 2018 is shown to demonstrate the usefulness of our system through recent disease-related events. Through the "Topics" and "New Topics," pages we can increase the rate of information dissemination for various disease-related topics and find other related information. In addition, many of these events are overshadowed in media by high-profile events such as celebrity and political scandals; however, our systems can capture them.

**Table 4:** Extracted disease related topics list.

Date	Topics page	New Topics page
Sep 28, 2017	Hygienic band	Hygienic band, Volcano
Oct 1, 2017	Red imported fire ant	Red imported fire ant–Incheon
Oct 11, 2017	AIDS	AIDS, Tuberculosis
Oct 24, 2017	Pseudomonas aeruginosa	Pseudomonas aeruginosa
Nov 4, 2017	Legionella	Legionella–Thermal spring
Nov 8, 2017	Egg and Fipronil	Egg and Fipronil
Dec 12, 2017	Duck	Duck–Netherlands
Dec 17, 2017	Newborn baby	Newborn baby–Mokdong
Dec 24, 2017	Bacillus anthracis	Bacillus anthracis, Rota virus
Jan 7, 2018	Pig	Pig–Africa
Jan 9, 2018	Cosmetics	Cosmetics, Newborn baby
Jan 17, 2018	Fine dust	Fine dust
Jan 26, 2018	Hospital fire	Hospital fire–Milyang
Feb 4, 2018	Malaria	Malaria
Feb 23, 2018	Asbestos	Asbestos
Feb 28, 2018	Typhoid	Sexual violence
Mar 7, 2018	Hepatitis	Glaucoma
Mar 9, 2018	Game	Game
Mar 11, 2018	Hepatitis, Game	Fine dust
Mar 12, 2018	Pigeon Corporation	Pigeon Corporation
Mar 13, 2018	Pigeon Corporation	Rota virus
Mar 14, 2018	Hepatitis	Chungchengbuk-do–Avian Influenza
Mar 15, 2018	Forest fire	Shellfish toxin
Mar 16, 2018	Hepatitis	Rabies virus–Thailand
Mar 17, 2018	Hepatitis	Gyeonggi-do–Avian Influenza
Mar 18, 2018	Prevention of epidemics	Pyeongtaek city–Avian Influenza
Mar 19, 2018	Vaccine	Cosmetics
Mar 20, 2018	AmorePacific Corporation–Cosmetics	AmorePacific Corporation–Cosmetics
Mar 21, 2018	Vaccine	Plastic–Water bottles
Mar 22, 2018	Tuberculosis	Tuberculosis
Mar 23, 2018	Tuberculosis	Shellfish toxin–Mussel
Mar 25, 2018	Tuberculosis	Fine dust–Mask
Mar 26, 2018	Fine dust–Mask	Yellow dust
Mar 27, 2018	Foot-and-mouth disease–Pig	Foot-and-mouth disease–Pig
Mar 29, 2018	Foot-and-mouth disease–Pig	Vibrio Vulnificus Septicemia–Yeosu

## Conclusion

DiTeX is the world's first system that extracts important disease-related topics from web-based data. Web-based data are important resources in disease surveillance systems, but it still brings unresolved issues. Online news sources interrupt the extraction of disease-related data by repeatedly using major keywords. SNS data is generated by many unspecified users, making it very difficult to extract accurate information owing to noise such as typos, repetition of meaningless words, and spam. In order to solve these problems, we successfully extracted data with minimal duplication using string similarity checks and converted incomplete sentences into complete sentences through open-source NLP to facilitate disease-related topic extraction. Our system extracts the most important disease-related topics using CCA, an advanced ranking algorithm. Finally, we develop our system for a web environment accessible from a variety of platforms. It allows general public to access and search for important disease-related topics at any time. Moreover it will be valuable research resources for public disease specialists because it provides not only current but also long term information.

## Limitations and research agenda

We have three further ongoing works. The first is the ambiguity of the word. A representative example is "Virus". "Virus" can be used in a biological mean like "Zika virus", it can be also used in the computer like "Computer virus", and it can be used as "happy virus" on Twitter [17]. A “happy virus” means a person who makes you smile no matter what. The data crawler collects web-based data through search keywords, so it is difficult to identify the ambiguity of “Virus”. We solve the ambiguity through machine learning. We combine DiTeX with the artificial neural network mixed Word2Vec [61], which allows the computer to understand human languages, so that only disease-related data can be extracted. The second is the multi-national language support. DiTeX currently supports Korean language, so there is some limitation on collecting multi-language data. Therefore, we expand the collection range of the data crawler and develop a multilingual NLP. Finally, DiTeX extracts disease-related topics by synthesizing the data collected the day before, so it has a problem that it cannot respond immediately when an infectious disease occurs. In order to solve the real-time problem, we develop DiTeX that can extract disease-related topics every hour. DiTeX will be able to extract disease-related topics in real-time. We are trying to expand the utility of DiTeX to surpass the three limitations. We believe that DiTeX can be the system that can be used globally and can make great helps for public health in near future.

## References

[1] Carneiro HA, Mylonakis E. Google trends: a web-based tool for real-time surveillance of disease outbreaks. Clin Infect Dis. 2009;49: 1557–1564. 10.1086/630200 19845471

[2] Santillana M, Nguyen AT, Louie T, Zink A, Gray J, Sung I, et al Cloud-based electronic health records for real-time, region-specific influenza surveillance. Sci Rep. 2016;6: 25732 10.1038/srep25732 27165494 PMC4863169

[3] Yang W, Olson DR, Shaman J. Forecasting influenza outbreaks in boroughs and neighborhoods of New York City. PLoS Comput Biol. 2016;12: e1005201 10.1371/journal.pcbi.1005201 27855155 PMC5113861

[4] Yuan Q, Nsoesie EO, Lv B, Peng G, Chunara R, Brownstein JS. Monitoring influenza epidemics in china with search query from baidu. PloS One. 2013;8: e64323 10.1371/journal.pone.0064323 23750192 PMC3667820

[5] Xu Q, Gel YR, Ramirez LLR, Nezafati K, Zhang Q, Tsui K-L. Forecasting influenza in Hong Kong with Google search queries and statistical model fusion. PloS One. 2017;12: e0176690 10.1371/journal.pone.0176690 28464015 PMC5413039

[6] Hiltz SR, Turoff M. Structuring computer-mediated communication systems to avoid information overload. Commun ACM. 1985;28: 680–689.

[7] Laskin DM. Dealing with information overload. J Oral Maxillofac Surg. 1994;52: 661 8006728 10.1016/0278-2391(94)90473-1

[8] Earle PS, Bowden DC, Guy M. Twitter earthquake detection: earthquake monitoring in a social world. Ann Geophys. 2012;54.

[9] Sakaki T, Okazaki M, Matsuo Y. Earthquake shakes Twitter users: real-time event detection by social sensors. Proceedings of the 19th international conference on World wide web. ACM; 2010. pp. 851–860.

[10] Park CS. Does Twitter motivate involvement in politics? Tweeting, opinion leadership, and political engagement. Comput Hum Behav. 2013;29: 1641–1648.

[11] Tumasjan A, Sprenger TO, Sandner PG, Welpe IM. Election forecasts with Twitter: How 140 characters reflect the political landscape. Soc Sci Comput Rev. 2011;29: 402–418.

[12] Tumasjan A, Sprenger TO, Sandner PG, Welpe IM. Predicting elections with twitter: What 140 characters reveal about political sentiment. Icwsm. 2010;10: 178–185.

[13] Dredze M, Paul MJ, Bergsma S, Tran H. Carmen: A twitter geolocation system with applications to public health. AAAI workshop on expanding the boundaries of health informatics using AI (HIAI). 2013 p. 45.

[14] Heaivilin N, Gerbert B, Page JE, Gibbs JL. Public health surveillance of dental pain via Twitter. J Dent Res. 2011;90: 1047–1051. 10.1177/0022034511415273 21768306 PMC3169887

[15] Paul MJ, Dredze M. You are what you Tweet: Analyzing Twitter for public health. Icwsm. 2011;20: 265–272.

[16] Paul MJ, Dredze M. A model for mining public health topics from Twitter. Health (N Y). 2012;11: 16–6.

[17] Jang B, Yoon J. Characteristics Analysis of Data from News and Social Network Services IEEE Access 2018;

[18] Khan AZ, Atique M, Thakare VM. Combining lexicon-based and learning-based methods for Twitter sentiment analysis. Int J Electron Commun Soft Comput Sci Eng IJECSCSE. 2015; 89.

[19] Kwak H, Lee C, Park H, Moon S. What is Twitter, a social network or a news media? Proceedings of the 19th international conference on World wide web. ACM; 2010. pp. 591–600.

[20] Mendoza M, Poblete B, Castillo C. Twitter Under Crisis: Can we trust what we RT? Proceedings of the first workshop on social media analytics. ACM; 2010 pp. 71–79.

[21] Mitchell L, Frank MR, Harris KD, Dodds PS, Danforth CM. The geography of happiness: Connecting twitter sentiment and expression, demographics, and objective characteristics of place. PloS One. 2013;8: e64417 10.1371/journal.pone.0064417 23734200 PMC3667195

[22] Sloan L, Morgan J, Burnap P, Williams M. Who tweets? Deriving the demographic characteristics of age, occupation and social class from Twitter user meta-data. PloS One. 2015;10: e0115545 10.1371/journal.pone.0115545 25729900 PMC4346393

[23] Freifeld CC, Mandl KD, Reis BY, Brownstein JS. HealthMap: global infectious disease monitoring through automated classification and visualization of Internet media reports. J Am Med Inform Assoc. 2008;15: 150–157. 10.1197/jamia.M2544 18096908 PMC2274789

[24] Collier N, Doan S, Kawazoe A, Goodwin RM, Conway M, Tateno Y, et al BioCaster: detecting public health rumors with a Web-based text mining system. Bioinformatics. 2008;24: 2940–2941. 10.1093/bioinformatics/btn534 18922806 PMC2639299

[25] Keller M, Blench M, Tolentino H, Freifeld CC, Mandl KD, Mawudeku A, et al Use of unstructured event-based reports for global infectious disease surveillance. Emerg Infect Dis. 2009;15: 689 10.3201/eid1505.081114 19402953 PMC2687026

[26] Lu Y, Zhang P, Liu J, Li J, Deng S. Health-related hot topic detection in online communities using text clustering. Plos One. 2013;8: e56221 10.1371/journal.pone.0056221 23457530 PMC3574139

[27] Prier KW, Smith MS, Giraud-Carrier C, Hanson CL. Identifying health-related topics on twitter International conference on social computing, behavioral-cultural modeling, and prediction. Springer; 2011 pp. 18–25.

[28] Bian J, Topaloglu U, Yu F. Towards large-scale twitter mining for drug-related adverse events Proceedings of the 2012 international workshop on Smart health and wellbeing. ACM; 2012 pp. 25–32. 10.1145/2389707.2389713 PMC5619871 28967001

[29] Salton G, Buckley C. Term-weighting approaches in automatic text retrieval. Inf Process Manag. 1988;24: 513–523.

[30] Witten IH, Moffat A, Bell TC. Managing gigabytes: compressing and indexing documents and images Morgan Kaufmann; 1999.

[31] Singhal A, Buckley C, Mitra M. Pivoted document length normalization ACM SIGIR Forum. ACM; 2017 pp. 176–184.

[32] Buckley C, Salton G, Allan J, Singhal A. Automatic query expansion using SMART: TREC 3. NIST Spec Publ Sp. 1995; 69–69.

[33] Robertson S, Zaragoza H. The probabilistic relevance framework: BM25 and beyond. Found Trends® Inf Retr. 2009;3: 333–389.

[34] Robertson SE, Walker S, Jones S, Hancock-Beaulieu MM, Gatford M. Okapi at TREC-3. Nist Spec Publ Sp. 1995;109: 109.

[35] Allan J, Connell ME, Croft WB, Feng F-F, Fisher D, Li X. Inquery and trec-9 MASSACHUSETTS UNIV AMHERST CENTER FOR INTELLIGENT INFORMATION RETRIEVAL; 2000.

[36] Ramos J. Using tf-idf to determine word relevance in document queries. Proceedings of the first instructional conference on machine learning. 2003. pp. 133–142.

[37] de Almeida HM, Gonçalves MA, Cristo M, Calado P. A combined component approach for finding collection-adapted ranking functions based on genetic programming. Proceedings of the 30th annual international ACM SIGIR conference on Research and development in information retrieval. ACM; 2007. pp. 399–406.

[38] Koza JR. Genetic programming as a means for programming computers by natural selection. Stat Comput. 1994;4: 87–112.

[39] Momjian B. PostgreSQL: introduction and concepts Addison-Wesley New York; 2001.

[40] Ayres DL, Darling A, Zwickl DJ, Beerli P, Holder MT, Lewis PO, et al BEAGLE: an application programming interface and high-performance computing library for statistical phylogenetics. Syst Biol. 2011;61: 170–173. 10.1093/sysbio/syr100 21963610 PMC3243739

[41] Bernhard D, Gurevych I. Answering learners’ questions by retrieving question paraphrases from social Q&A sites Proceedings of the third workshop on innovative use of NLP for building educational applications. Association for Computational Linguistics; 2008 pp. 44–52.

[42] Ong SP, Cholia S, Jain A, Brafman M, Gunter D, Ceder G, et al The Materials Application Programming Interface (API): A simple, flexible and efficient API for materials data based on REpresentational State Transfer (REST) principles. Comput Mater Sci. 2015;97: 209–215.

[43] Ihrig CJ. Javascript object notation Pro Node js for Developers. Springer; 2013 pp. 263–270.

[44] Protocol HT, Berners-Lee T, Draft CI. Hypertext Transfer Protocol.

[45] Johnson R, Hoeller J, Arendsen A, Thomas R. Professional Java development with the Spring framework John Wiley & Sons; 2009.

[46] Pimentel V, Nickerson BG. Communicating and displaying real-time data with websocket. IEEE Internet Comput. 2012;16: 45–53.

[47] Bucanek J. Model-view-controller pattern. Learn Object-C Java Dev. 2009; 353–402.

[48] Super Fast and Accurate string distance algorithm: Sift4. In: Siderite’s Blog [Internet]. [cited 19 Apr 2018]. Available: https://siderite.blogspot.com/2014/11/super-fast-and-accurate-string-distance.html

[49] Cohen W, Ravikumar P, Fienberg S. A comparison of string metrics for matching names and records. Kdd workshop on data cleaning and object consolidation. 2003 pp. 73–78.

[50] Wagner RA, Fischer MJ. The string-to-string correction problem. J ACM JACM. 1974;21: 168–173.

[51] Jeon H, Kim T. KoNLP: Korean NLP package. R Package Version 080 0. 2016;

[52] Gosling J. The Java language specification Addison-Wesley Professional; 2000.

[53] Odersky M, Altherr P, Cremet V, Emir B, Micheloud S, Mihaylov N, et al The Scala language specification. 2004.

[54] Bibeault B, Kats Y. jQuery in Action Dreamtech Press; 2008.

[55] van Wijngaarden T. Asynchronous JavaScript and XML.

[56] DataTables | Table plug-in for jQuery [Internet]. [cited 19 Apr 2018]. Available: https://datatables.net/

[57] Bostock M. D3. js. Data Driven Doc. 2012;492: 701.

[58] Downie N. Chart. js| Open source HTML5 Charts for your website. Chart Js. 2015;

[59] Halkidi M, Batistakis Y, Vazirgiannis M. On clustering validation techniques. J Intell Inf Syst. 2001;17: 107–145.

[60] Schmidt CO, Kohlmann T. When to use the odds ratio or the relative risk? Int J Public Health. 2008;53: 165–167. 19127890 10.1007/s00038-008-7068-3

[61] Goldberg Y, Levy O. word2vec explained: Deriving mikolov et al.’s negative-sampling word-embedding method. ArXiv Prepr ArXiv14023722. 2014;

